# Development and verification of the glycolysis-associated and immune-related prognosis signature for hepatocellular carcinoma

**DOI:** 10.3389/fgene.2022.955673

**Published:** 2022-10-04

**Authors:** Bo Hu, Chao Qu, Wei-Jun Qi, Cheng-Hao Liu, Dian-Rong Xiu

**Affiliations:** Department of General Surgery, Peking University Third Hospital, Beijing, China

**Keywords:** hepatocellular carcinoma, glycolysis, immune system, prognosis, metabolic reprogramming

## Abstract

**Background:** Hepatocellular carcinoma (HCC) refers to the malignant tumor associated with a high mortality rate. This work focused on identifying a robust tumor glycolysis-immune-related gene signature to facilitate the prognosis prediction of HCC cases.

**Methods:** This work adopted t-SNE algorithms for predicting glycolysis status in accordance with The Cancer Genome Atlas (TCGA)-derived cohort transcriptome profiles. In addition, the Cox regression model was utilized together with LASSO to identify prognosis-related genes (PRGs). In addition, the results were externally validated with the International Cancer Genome Consortium (ICGC) cohort.

**Results:** Accordingly, the glycolysis-immune-related gene signature, which consisted of seven genes, *PSRC1*, *CHORDC1*, *KPNA2*, *CDCA8*, *G6PD*, *NEIL3*, and *EZH2,* was constructed based on TCGA-HCC patients. Under a range of circumstances, low-risk patients had extended overall survival (OS) compared with high-risk patients. Additionally, the developed gene signature acted as the independent factor, which was significantly associated with clinical stage, grade, portal vein invasion, and intrahepatic vein invasion among HCC cases. In addition, as revealed by the receiver operating characteristic (ROC) curve, the model showed high efficiency. Moreover, the different glycolysis and immune statuses between the two groups were further revealed by functional analysis.

**Conclusion:** Our as-constructed prognosis prediction model contributes to HCC risk stratification.

## Introduction

Liver cancer (LC), dominated by hepatocellular carcinoma (HCC), ranks fourth among death-related causes (McGlynn et al., 2021). At present, some effective treatments are available to treat early HCC, which include surgery, liver transplantation, and radiofrequency ablation (RFA) (Yang et al., 2019). Nonetheless, considering the non-specific HCC physical features and symptoms, 80% of patients are deprived of the chance of radical treatment when they are diagnosed (Zongyi and Xiaowu, 2020). Recently, some immune checkpoint inhibitors (ICIs), such as anti-cytotoxic T-lymphocyte-associated protein 4 (anti-CTLA-4), anti-PD1 ligand (anti-PD-L1), and anti-programmed death-1 (anti-PD1) monoclonal antibodies (mAbs) (Hu et al., 2019), have demonstrated good prognosis among some advanced HCC patients. In conclusion, it is urgently needed for exploring the HCC characteristics for developing new therapeutic approaches.

The tumor microenvironment (TME) shows the characteristics of acidity, hypoxia, and nutrient deficiency, leading to abnormal metabolism of tumor cells and those adjacent to the stromal cells, finally promoting cancer migration, growth, and survival (Beloribi-Djefaflia et al., 2016; Ruocco et al., 2019). The Warburg effect is one of the most notable types of metabolic reprogramming, in which glycolysis increases among tumor cells and serves as the metabolic marker for almost every cancer cell, and it is featured by the excess transformation of glucose into lactate (Liberti and Locasale, 2016). Glycolysis has been proven to promote tumor progression in HCC and is an early attractive target for cancer treatment (Vander Heiden, 2011). Hamaguchi *et al.* demonstrated that the HIF-1α-activated glycolysis module was associated with the aggressiveness of HCC (Hamaguchi et al., 2008). A study conducted by Xu *et al.* also suggested that tumor glycolysis was inhibited by chrysin, which then induced apoptosis of HCC by targeting hexokinase-2 (Xu et al., 2017). Moreover, previous studies also highlight that glycolysis affects immunity. T-cell cytotoxicity and trafficking have been illustrated to be damaged in glycolytic cancers, and glycolysis suppression will enhance the anticancer effect of tumor-active T cells (Cascone et al., 2018). Lim *et al.* also pointed out that EGFR signaling enhances aerobic glycolysis to promote tumor growth and immune escape (Lim et al., 2016). However, the relation of glycolysis with immunity and their impact on the prognosis of HCC remains largely unclear.

In this work, it was hypothesized that the association of glycolysis with immune status had a certain prognostic value of HCC. By systematically analyzing glycolysis and immune status as the existing clinicopathological features and staging systems, new prognostic features were constructed to improve the prognosis of HCC.

## Materials and methods

### Data extraction and mRNA profile mining

This work acquired expression patterns of class 3 messenger RNA (mRNA), along with associated clinical data in 374 HCC as well as 50 normal subjects in The Cancer Genome Atlas (TCGA) database (https://cancergenome.nih.gov). Patients whose survival was ≤ 30 days or those who had no survival data were excluded from the present work because they might have died from lethal complications (including intracranial infection, hemorrhage, or heart failure (HF)) but not HCC. To validate our results, transcriptional patterns and clinical information were obtained from HCC cases in the International Cancer Genome Consortium (ICGC) database (https://icgc.org/). Finally, this work screened 203 HCC specimens that had sufficient mRNA expression profiles as well as matching clinical profiles to carry out further analysis.

### Glycolytic status and glycolysis-related differentially expressed gene identification

This work utilized the t-distributed Stochastic Neighbor Embedding (t-SNE) algorithm to identify the different glycolytic states (Cieslak et al., 2019). The t-SNE is an unsupervised non-parametric method, which allows the division or condensation of patients into several different clusters based on the provided markers or features. In this study, the glycolytic marker gene set (*n* = 200) was obtained from the molecular signature database (MSigDB version 6.0). Moreover, overall survival (OS) between the different clusters derived from the t-SNE algorithm was analyzed. Subsequently, two clusters with discrete OS were selected to assess the glycolysis status. Also, differentially expressed genes (DEGs) between the two groups were identified using the R3.6.1 “limma” algorithm (Phipson et al., 2016) (https://www.r-project.org/). In addition, after adjusting for false discovery rate (FDR), this work selected genes of *p* < 0.01 and absolute fold change (FC) > 2 as the glycolytic DEGs.

### Development and validation of the glycolysis-related prognosis model

Afterward, this work built the glycolysis-associated prognostic model by univariate, multivariate regression, and the least absolute shrinkage and selection operator (LASSO) for predicting the OS of HCC. To avoid overfitting and also to remove the closely related genes, this work adopted LASSO analysis and extracted significant genes based on univariate regression. Thereafter, related gene contributions to the prediction of prognosis were assessed by multivariate Cox regression. Additionally, gene level was multiplied by linear combination regression coefficients obtained from multivariate analysis to determine the risk score. In line with the median risk score value, the patients were later categorized into low- and high-risk groups.

Afterward, this work drew the Kaplan–Meier (K-M) curves using “survminer” and “survival” of the R package. Later, the time‐dependent receiver operating characteristic (t-ROC) curves were drawn for evaluating the efficiency of our signature in predicting prognosis based on TCGA and ICGC databases by identifying the area under the curve (AUC) values (Blanche et al., 2013). Thereafter, this study carried out univariate and multivariate Cox proportional hazards regression analyses for confirming our model efficiency in independently predicting liver fibrosis prognosis by using traditional clinical factors like age, sex, clinical stage, the status of hepatitis virus infection, liver fibrosis, bile duct invasion, portal vein invasion, and intrahepatic vein invasion in the HCC cohort derived from ICGC database. To be specific, liver fibrosis, bile duct invasion, portal vein invasion, and intrahepatic vein invasion were selected from the HCC cohort of the ICGC database, whereas age, sex, T-stage, clinical stage, tumor grade, vascular invasion, albumin level, platelet level, and alpha-fetoprotein (AFP) level were selected from HCC cohort of the TCGA database.

Furthermore, this work conducted principal component analysis (PCA) to reduce dimensions, so as to identify different synthetic factors to explore our model’s stratification performance (Raychaudhuri et al., 2000). The present work utilized limma (Matthew et al., 2015) and scatterplot3d (Koutecký, 2015) packages for PCA. To better examine glycolytic status in low- and high-risk patients obtained based on those constructed signatures, this work conducted gene set enrichment analysis (GESA) using JAVA (https://www.broadinstitute.org/gsea). *p* < 0.05 and FDR q < 0.25 were selected to be the significance levels.

### Relationships between the glycolysis-related prognosis prediction model and immunocyte infiltration

Previous studies have shown that glycolysis has a certain impact on immune cells and tumor progression (Cascone et al., 2018; Chen et al., 2019), and they contribute to the exploration of the link of our constructed prognosis model within tumor-infiltrating immune cells (TIICs) in HCC. TIMER database is also the online platform used for the systemic assessment of diverse TIICs’ influence on a variety of different cancers, and for the analysis and visualization of TIIC abundances (Robin et al., 2011). There are 10,009 TCCA samples of 23 cancers in TIMER for predicting the abundances of six TIIC subtypes, namely, CD4 T cells, CD8 T cells, B cells, neutrophils, macrophages, and dendritic cells (DCs). Therefore, this study utilized TIMER for identifying the relation of TIICs with additional factors. This work obtained TIICs infiltration degrees in HCC cases derived from TCGA and determined the relation of our established prognosis model with TIICs infiltration degrees. Moreover, this work also examined the association between the levels of signature genes and TIICs abundances in HCC *via* the online gene modules. Furthermore, ESTIMATE (Yoshihara et al., 2013) was adopted to measure the tumor purity, TIICs infiltration degree (immune score), and stromal content (stromal score) in low- and high-risk HCC patients.

### Immunohistochemistry

According to the results of bioinformatics analysis, the protein expression of genes that are included in the glycolysis-associated and immune-related prognosis signature were validated by immunohistochemistry (IHC) using fifteen pairs of matched HCC and para-carcinoma tissue obtained from our center. Anti-model gene antibodies were purchased from Abcam (Cambridge, MA, United States). The specimens were paraffin-embedded and sliced into 4-μm sections. The sections were dewaxed and treated with 3% hydrogen peroxide for 10 min to block the endogenous peroxidase. The sections were washed twice with distilled water and boiled in 0.01 M citrate buffer (pH 6.0) for heat-induced antigen retrieval. Sections were then washed thrice with PBS and blocked with 5% goat serum (Zhongshan Golden Bridge Biotech, Beijing, China) at room temperature for 30 min. Subsequently, sections were incubated with the appropriate primary antibody for 1 h at 37°C, washed thrice (5 min) with TBS-Tween® 20 (TBST), incubated with HPR-conjugated anti-human secondary antibody for 30 min at room temperature, washed thrice (5 min) with PBS, and developed with freshly prepared DAB for 10 min. Sections were washed with tap water, counterstained with hematoxylin, and mounted using neutral gum (Zhongshan Golden Bridge Biotech.) onto the slides. The stained sections were scanned using an automated IHC high-definition scanner (Jiangfeng Electronics, Zhejiang, China). The positive expression was detected as brown in the cytoplasm/nuclei/membrane. The data were analyzed using ImageJ and the graph was generated using GraphPad prism 5.0.

### Statistical analysis

R4.2.0 was adopted for statistical analysis. Qualitative variables were investigated by Fisher’s exact test and Pearson’s chi-square test. Meanwhile, this work also utilized the “Rtsne” package in R for performing the t-SNE algorithm by a nonlinear down-scale. In addition, the present study also utilized the “estimation” package for inputting immune scores, whereas the “glmnet” package for developing the Lasso Cox regression model. Unless otherwise stated, *p* < 0.05 indicated that a difference was statistically significant.

## Results

### Glycolysis-related differentially expressed gene identification within hepatocellular carcinoma

**FIGURE 1:**
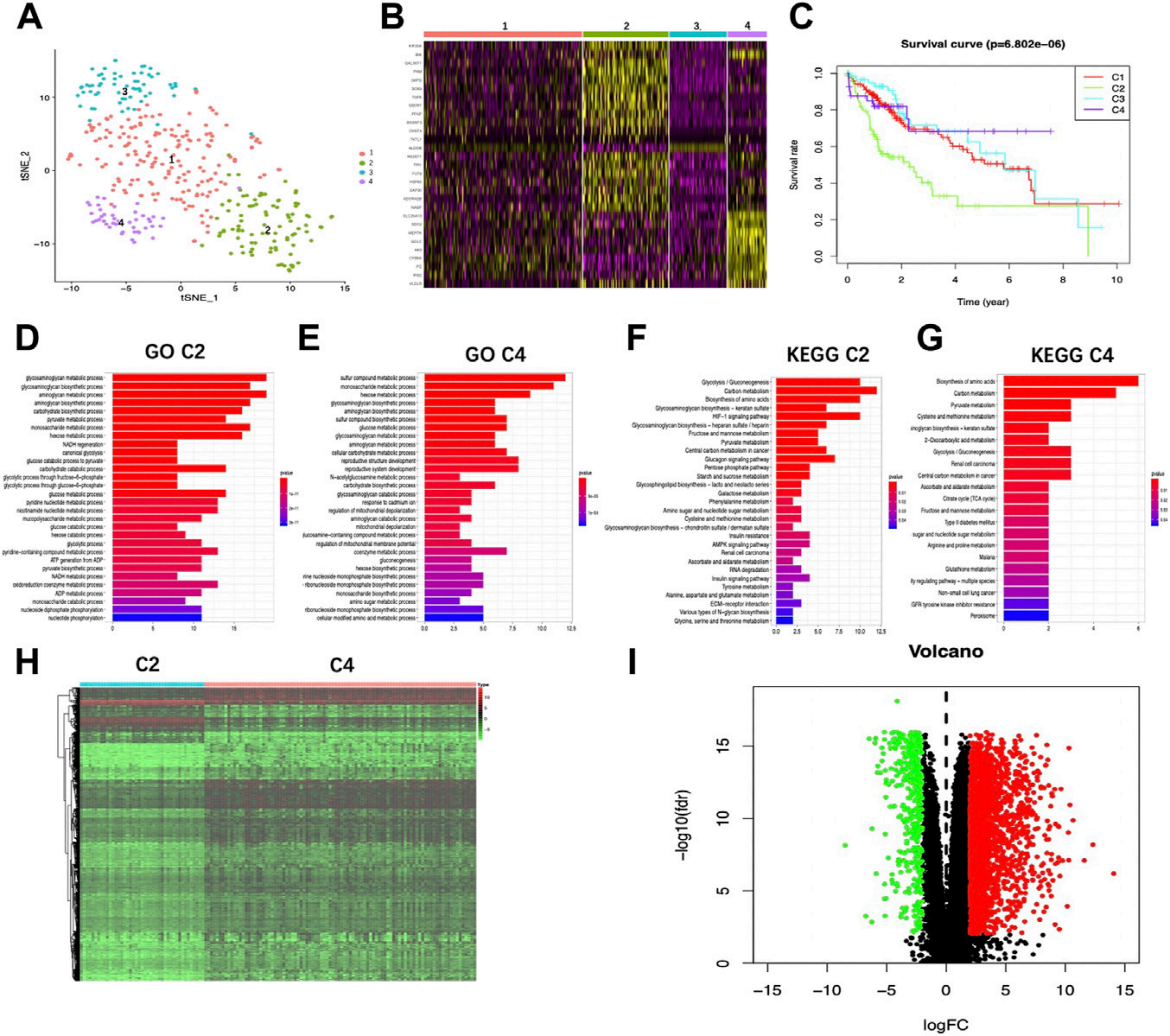
Glycolysis status and glycolysis associated DEG determination. **(A)** Dot plot showing the four different clusters distinguished using t-SNE algorithm based on 200 glycolysis-associated marker genes. **(B)** Heatmap showing the marker genes of the four clusters. **(C)** K-M plot showing patient OS in four clusters. **(D,E)** GO-BP terms enriched by marker genes of Cluster2 and Cluster4, respectively. **(F,G)** KEGG pathways enriched by marker genes of Cluster2 and Cluster4, respectively. Heatmap **(H)** as well as volcano plot **(I)** displaying the glycolysis associated DEGs in HCC compared with healthy samples. Red and green dots stand for DEGs with up-regulation and down-regulation, separately, whereas black dots indicate genes with no differential expression. T, tumor; N, normal tissue.

Using an expression matrix containing 200 glycolytic marker genes based on MSigDB version 6.0, this work calculated the Euclidean distance (ED) of two discovery cohort cases and clustered them into 2D points by t-SNE, the nonlinear dimensionality reduction algorithm. A total of four clusters were then identified in this study, thereafter, patients were assigned to the closest ED (Figure 1A). In total, 174, 94, 63, and 43 TCGA-HCC cases were collected into those four clusters (cluster 1–4), separately. Upon comparison of survival rates, there existed a significant difference between the four clusters (*p* < 0.05). Typically, cases in cluster 4 showed the best 5-year survival, while those in cluster 2 exhibited the worst prognosis (Figure 1B), indicating the highest and lowest glycolytic statuses of cluster 2 and cluster 4, respectively. In the meantime, Figure 1C displays a heatmap showing marker genes among the four clusters. Clearly, for cluster 2, its marker genes were mostly associated with certain glycolysis-associated Gene Oncology (GO) terms belonging to the biological process (BP) category, including “glycolytic process” and “canonical glycolysis,” together with “glycolytic process *via* glucose−6−phosphate” (Figure 1D; Supplementary Table S1). Additionally, according to Kyoto Encyclopedia of Genes and Genomes (KEGG) results, marker genes were also enriched in “glycolysis/gluconeogenesis”, “central carbon metabolism in cancer” and “AMPK pathway” (Figure 1E; Supplementary Table S2). GO and KEGG results concerning the marker genes of cluster 4 were obtained as well (Figures 1F,G; Supplementary Tables S3, S4). Afterward, the edgeR algorithm was employed for identifying altogether 2,777 DEGs in cluster 2 versus cluster 4, including 2372 with upregulation whereas 405 showed downregulation (Figures 1H,I).

### Development of a glycolysis-associated signature and assessment of the prediction performance

**FIGURE 2:**
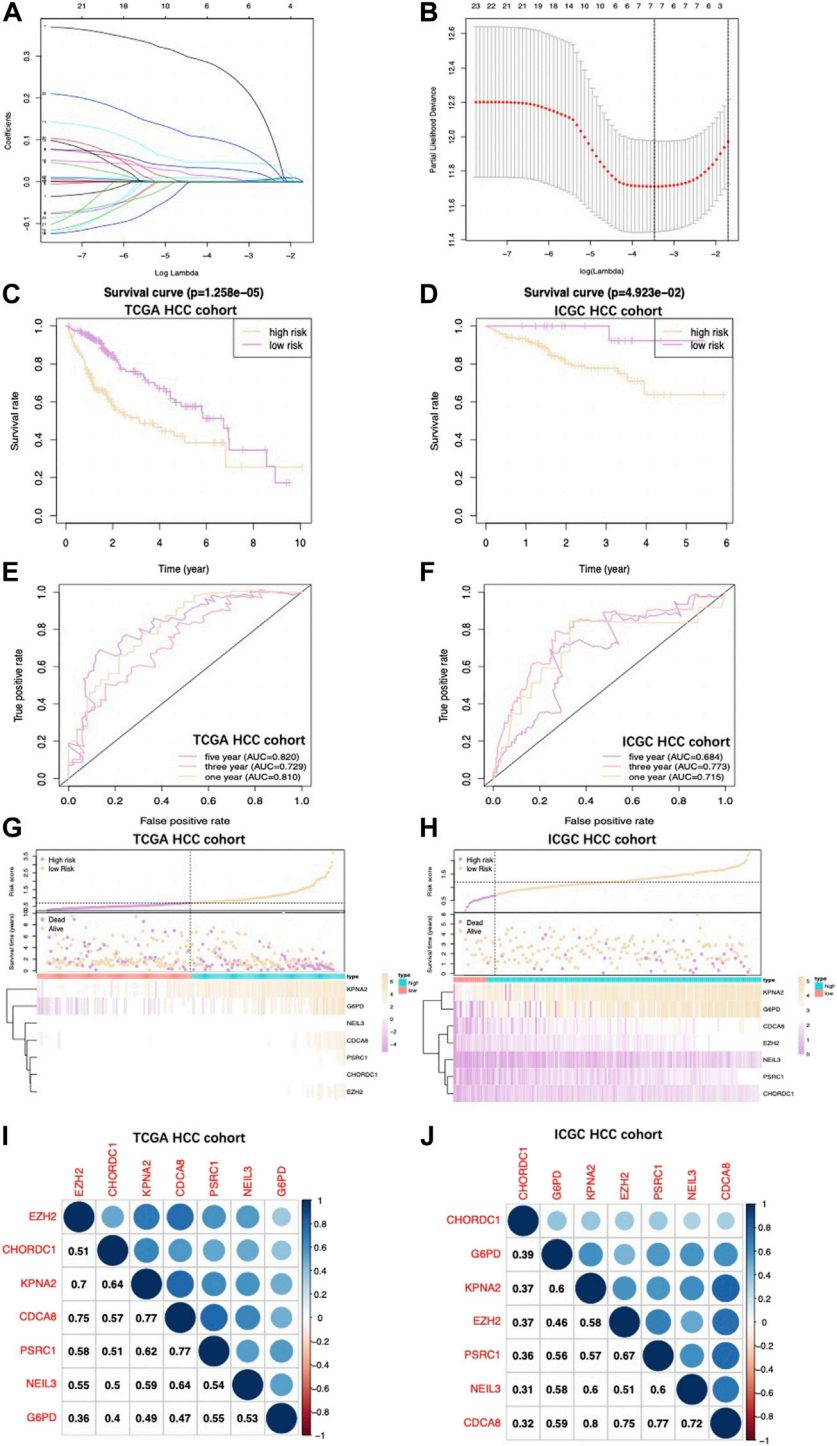
**(A)** Coefficient profiles obtained by LASSO analysis. **(B)** Screening for tuning parameter (lambda) based on the LASSO model through 10-fold cross-validation according to the minimal standards of OS, where log (lambda) is presented in the lower *X*-axis, while the mean OS gene quantity is shown in the upper *X*-axis, and deviance error of partial likelihood is presented in *Y*-axis. Red dots stand for mean deviance of partial likelihood of the model with specific lambda, whereas the vertical bars stand for upper/lower limits of deviance errors of partial likelihood. Vertical dotted lines in black define the optimal lambda value with the optimal fit. Patient survival curves for two HCC groups from TCGA **(C)** together with those from ICGC **(D)**. High‐risk group had shortened OS. **(E)**, **(F)** The survival‐dependent ROC curves validated by 1-, 3-, and 5-year prognostic values for the prognostic index of TCGA and ICGC, separately. Risk score, OS, and gene expression distributions of **(G)** TCGA and **(H)** ICGC databases. Risk score, OS together with heatmap for seven gene levels in two groups are displayed in the figure upside down. The correlations of seven genes in the **(I)** TCGA database and **(J)** ICGC database are shown.

**FIGURE 3:**
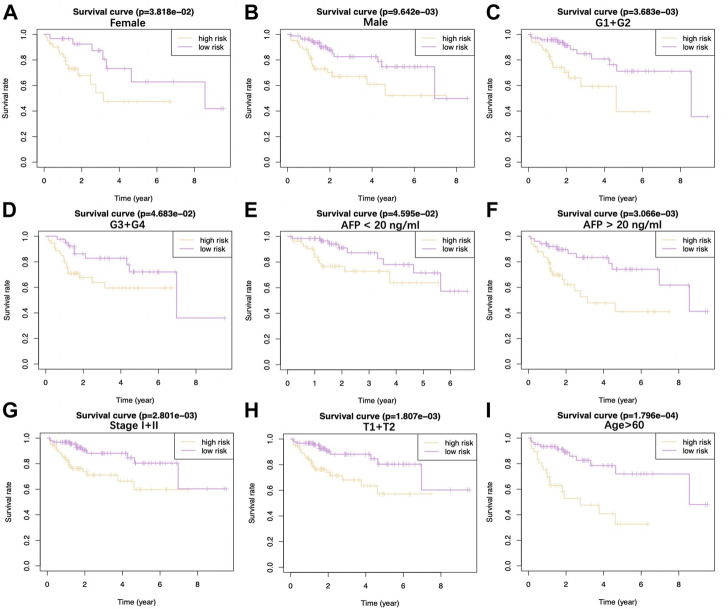
Difference in OS between two TCGA-HCC cohorts are presented after stratification by gender **(A**,**B)**, tumor grade **(C**,**D)**, AFP level **(E**,**F)**, clinical stage **(G)**, T stage **(H),** and age **(I)**.

Upon univariate, multivariate, and LASSO analysis, seven glycolysis-associated genes, namely, enhancer of zeste 2 polycomb repressive complex 2 subunit (*EZH2*); glucose-6-phosphate dehydrogenase (*G6PD*), cysteine and histidine-rich domain (CHORD) containing 1 (*CHORDC1*), nei endonuclease VIII-like 3 (*NEIL3*); proline/serine-rich coiled-coil 1 (*PSCR1*), karyopherin alpha 2 (*KPNA2*), and cell division cycle-associated *8* (*CDCA8*) were subsequently selected as DEGs for establishing the prognosis prediction signature. Later, the signature was used to categorize HCC cases into low- and high-risk groups (Figures 2A,B). Thereafter, the risk score was determined by this formula: risk score = [*PSRC1* level* (0.03075)] + [*CHORDC1* level * (0.26886)] + [*KPNA2* level* (0.00044)] + [*CDCA8* level* (0.04583)] + [*G6PD* level* (0.00854)] + [*EZH2* level* (0.01083)] + [*NEIL3* level* (0.09789)]. As revealed by the K-M curve (Figures 2C,D), the high-risk group had markedly reduced OS compared with the low-risk group from ICGC and TCGA databases. Apart from that, the AUC values of 1-, 3- and 5-years OS were calculated to be 0.810, 0.729, and 0.820 for the TCGA-HCC cohort, whereas 0.715, 0.773, and 0.684 for ICGC-HCC cohort, separately (Figures 2E,F). Additionally, gene expression, survival status, and risk score distributions were analyzed by our constructed glycolysis-associated signature from ICGC- and TCGA-HCC cohorts (Figures 2G,H). The correlations of the seven genes in ICHC- and TCGA-HCC cohorts are shown in Figures 2I,J. In the TCGA database, the Pearson correlation coefficients of *CDCA8* and *KPNA2*, *CDCA8*, and *PSRC1* reached 0.77, while in the ICGC database, the coefficients of *CDCA8* and *KPNA2* were 0.8. Moreover, the low-risk group was associated with a significantly superior prognostic outcome relative to the high-risk group with regard to subgroups classified by sex (female and male; Figures 3A,B), tumor grade (G1+G2 and G3+G4; Figures 3C,D), AFP content (< 20 and ≥ 20 ng/ml; Figures 3E,F), clinical stage (I + II; Figure 3G), T stage (T1+T2; Figure 3H), and age (> 60; Figure 3I) (*p* < 0.05). To better investigate whether our genetic signature was significant in independently predicting prognostic outcomes, univariate and the multivariate analysis was conducted. As a result, the risk score was the candidate factor independently predicting the prognosis of the TCGA-HCC cohort (hazard ratio HR: 3.401, 95% confidence intervals CIs: 2.078–5.566, *p* < 0.001) and ICGC-HCC cohort (HR: 7.768, 95% CIs: 2.302–26.204, *p* < 0.001) (Tables 1, 2, respectively).

**TABLE 1:** Univariate and multivariate regression on OS-related clinicopathological features for TCGA-HCC cases.

Variable	Univariate regression		Multivariate regression sis	
	HR (95% CI)	*P* value	HR (95% CI)	*P* value
Age (>60/≤60)	1.018 (0.995–1.041)	0.125	—	—
Gender (male/female)	0.601 (0.345–1.048)	0.073	—	—
Grade (G4/G3/G2/G1)	1.458 (0.997–2.133)	0.878	—	—
Stage (IV/III/II/I)	1.595 (1.184–2.148)	0.002^a^	1.801 (0.682–4.751)	0.235
T stage (T4/T3/T2/T1)	1.524 (1.134–2.047)	0.005^a^	0.878 (0.339–2.271)	0.789
Albumin (>3.5/≤3.5 g/dl)	0.964 (0.740–1.255)	0.784	—	—
Platelet (>250/≤250 × 10^9^/L)	0.999 (0.998–1.000)	0.288	—	—
AFP (>20/≤ 20 ng/ml)	1.000 (0.999–1.001)	0.435	—	—
Vascular invasion (macro/micro/none)	1.920 (1.263–2.920)	0.002^a^	1.554 (0.961–2.511)	0.072
Risk score	3.762(2.413–5.867)	<0.001^a^	3.401 (2.078–5.566)	<0.001^a^

a Statistical significance. AFP, alpha-fetoprotein; HR, hazard ratio; CI, confidence interval.

**TABLE 2:** Univariate and multivariate regression on OS-related clinicopathological features for ICGC-derived HCC cases.

Variable	Univariate regression		Multivariate regression	
	HR (95% CI)	*P* value	HR (95% CI)	*P* value
Age (>60/≤60)	1.021 (0.985–1.057)	0.255	—	—
Gender (male/female)	1.186 (0.538–2.617)	0.673	—	—
Hepatitis virus (none/infection)	0.929 (0.360–2.394)	0.878	—	—
Stage (IV/III/II/I)	1.373 (0.927–2.035)	0.114	—	—
Portal vein invasion (none/invasion)	1.076 (0.694–1.671)	0.741	—	—
Intrahepatic vein invasion (none/invasion)	2.243 (1.296–3.882)	0.004^a^	2.058 (0.896–4.733)	0.089
Bile duct invasion (none/invasion)	0.481 (0.066–3.516)	0.471	—	—
Fibrosis (none/fibrosis)	1.428 (0.195–10.439)	0.726	—	—
Risk score	7.048 (2.606–19.062)	<0.001^a^	7.767(2.302–26.204)	<0.001^a^

a Statistical significance. HR, hazard ratio; CI, confidence interval.

### Principal component analysis for verifying the signature stratification performance

**FIGURE 4:**
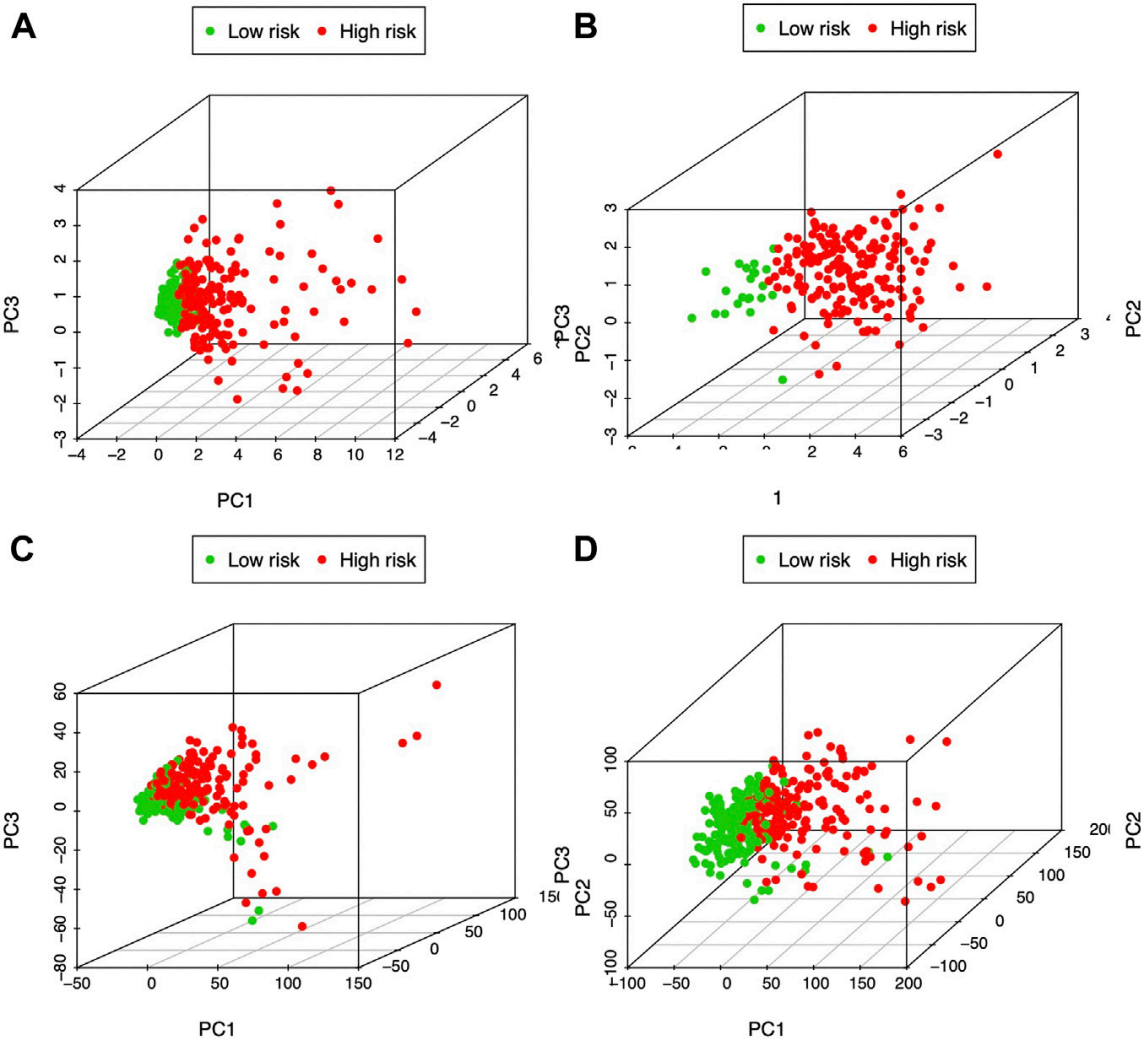
PCA of both groups according to the glycolysis-immune-associated prediction model in **(A)** TCGA and **(B)** ICGC databases, **(C)** glycolysis-associated DEGs in TCGA, and **(D)** all gene expression patterns in TCGA.

PCA was carried out to examine heterogeneity between the two groups according to our constructed glycolysis-associated signature (Figure 4A for TCGA patients and Figure 4B for ICGC patients), differently expressed glycolysis-associated genes in TCGA (Figure 4C), and all the gene expression patterns in TCGA (Figure 4D). Therefore, according to our model analysis, the two groups showed different distribution directions. Nonetheless, as shown in Figures 4C,D, an overlapping distribution between the two groups was observed, confirming the validity of our prognostic features for distinguishing the two groups.

### Association between our prognosis prediction model and the clinicopathological characteristics

**FIGURE 5:**
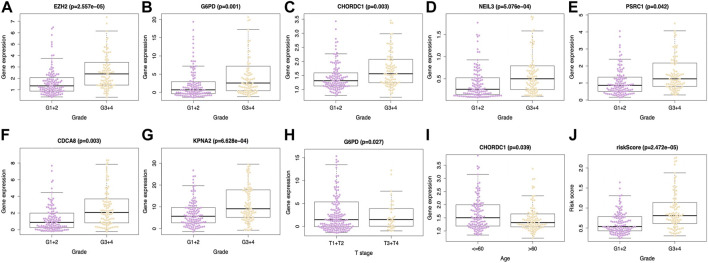
Relations between the prediction model and clinicopathological features based on TCGA database. Expression of **(A)** EZH2, **(B)** G6PD, **(C)** CHORDC1, **(D)** NEIL3, **(E)** PSRC1, **(F)** CDCA8 and **(G)** KPNA2 was associated with tumor grade. **(H)** G6PD expression was associated with T stage and **(I)** CHORDC1 expression was related to age. Moreover, **(J)** riskscore was linked with tumor grade. EZH2, enhancer of zeste 2 polycomb repressive complex 2 subunit; G6PD, glucose-6-phosphate dehydrogenase; CHORDC1, cysteine and histidine-rich domain (CHORD) containing 1; CDCA8, cell division cycle associated 8; NEIL3, nei endonuclease VIII-like 3; KPNA2, karyopherin alpha 2; PSRC1, proline/serine-rich coiled-coil 1.

This work included altogether 216 patients with complete data, such as age, sex, tumor grade, T-stage, clinical stage, albumin level, platelet level, AFP level, and vascular invasion from the TCGA-HCC cohort. All the tested signature genes were correlated with tumor grade (Figures 5A–G), among which, *G6PD* was associated with T-stage (Figure 5H). In addition, *CHORDC1* expression (Figure 5I) apparently increased among patients younger than 60 years old. Furthermore, the risk score value was significantly related to the patient histological grade (Figure 5J). From the ICGC database, all the seven model genes were correlated with clinical stage and intrahepatic vein invasion, whereas *CACD8*, *EZH2*, *G6PD*, *KPNA2*, and *PSRC1* were significantly related to portal vein invasion (Supplementary Figure S1). In addition, risk score was associated with clinical stage, portal vein invasion, and intrahepatic vein invasion (Supplementary Figure S1).

### Glycolysis status in low- and high-risk The Cancer Genome Atlas groups

**FIGURE 6:**
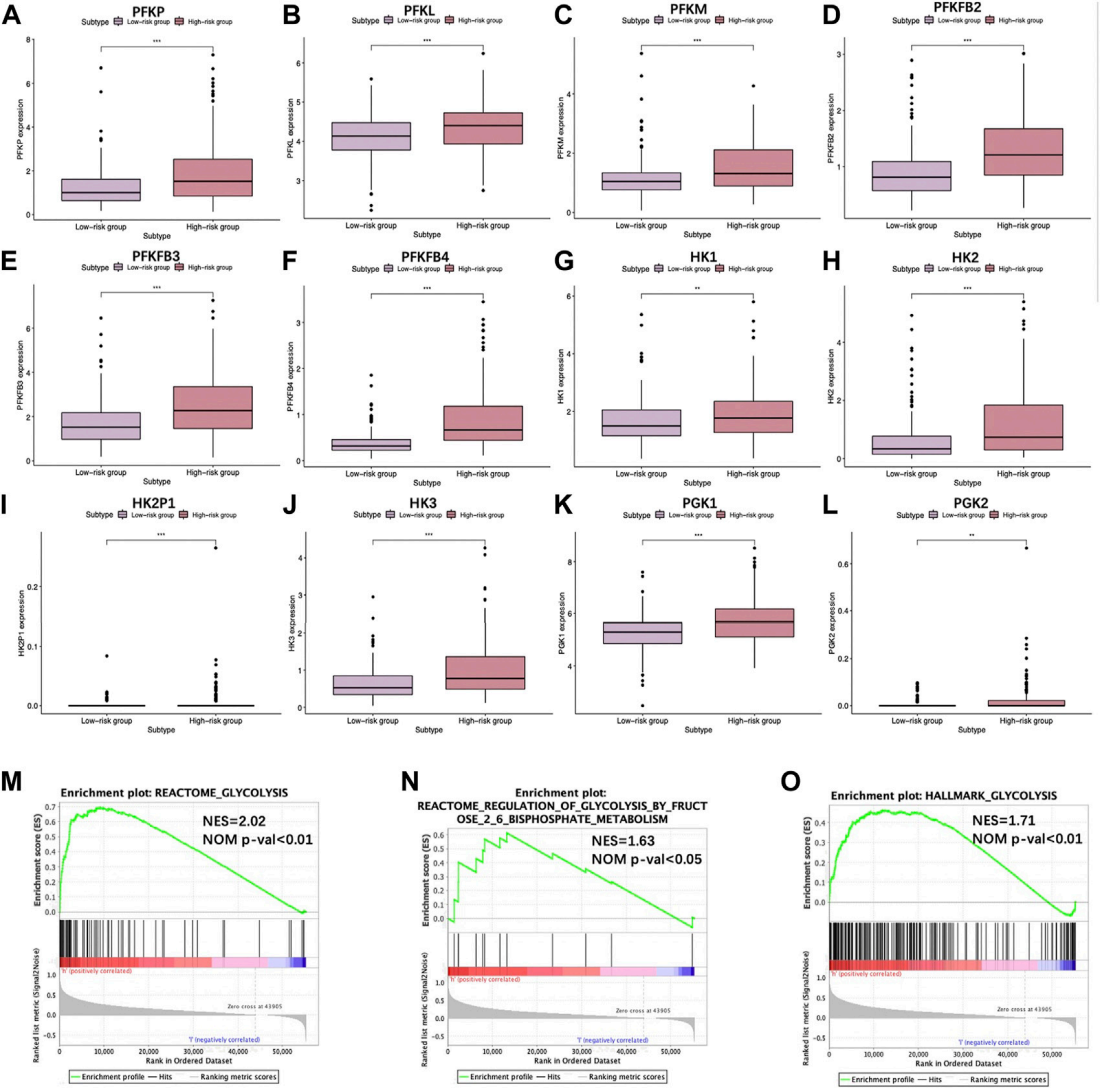
Scatter plots visualizing the significant differential expression of **(A)** phosphofructokinase liver (PFKL), **(B)** phosphofructokinase muscle (PFKM), **(C)** phosphofructokinase platelet (PFKP), **(D)** 6-phosphofructo-2-kinase/fructose-2,6-biphosphatase 2 (PFKFB2), **(E)** PFKFB3, **(F)** PFKFB4, **(G)** hexokinase 1 (HK1), **(H)** HK2, **(I)** HK2P1, **(J)** HK3, **(K)** phosphoglycerate kinase 1 (PGK1), and **(L)** PGK2 between two TCGA-HCC cohorts. **(M–O)** GSEA suggested that glycolysis-associated BPs were enriched by our constructed signature.

For validating diverse glycolysis statuses of two TCGA groups, the expression of key enzymes encoding genes in glycolysis between the two groups were analyzed, including hexokinase 1 (*HK1*), *HK2*, *HK2P1*, *HK3*, phosphofructokinase liver (*PFKL*), phosphofructokinase platelet (*PFKP*), phosphofructokinase muscle (*PFKM*), phosphoglycerate kinase 1 (*PGK1*), *PGK2*, 6-phosphofructo-2-kinase/fructose-2,6-biphosphatase 1 (*PFKFB1*), *PFKFB2*, *PFKFB3*, and *PFKFB4*. Noteworthily, except for *PFKFB1*, the remaining genes showed high expression levels in the high-risk group (*p* < 0.01) (Figures 6A–L), indicating a higher glycolysis level. Furthermore, GSEA was conducted, which suggested that DEGs of two groups were markedly enriched to three glycolysis-associated gene sets (including REACTOME_glycolysis M5113, REACTOME_regulation of glycolysis by fructose 2, 6 bisphosphate metabolism M27950 and HALLMARK_glycolysis M5937) (Figures 6M–O).

### Comparison of different tumor-infiltrating immune cell degrees between low- and high-risk The Cancer Genome Atlas-hepatocellular carcinoma cohorts

**FIGURE 7:**
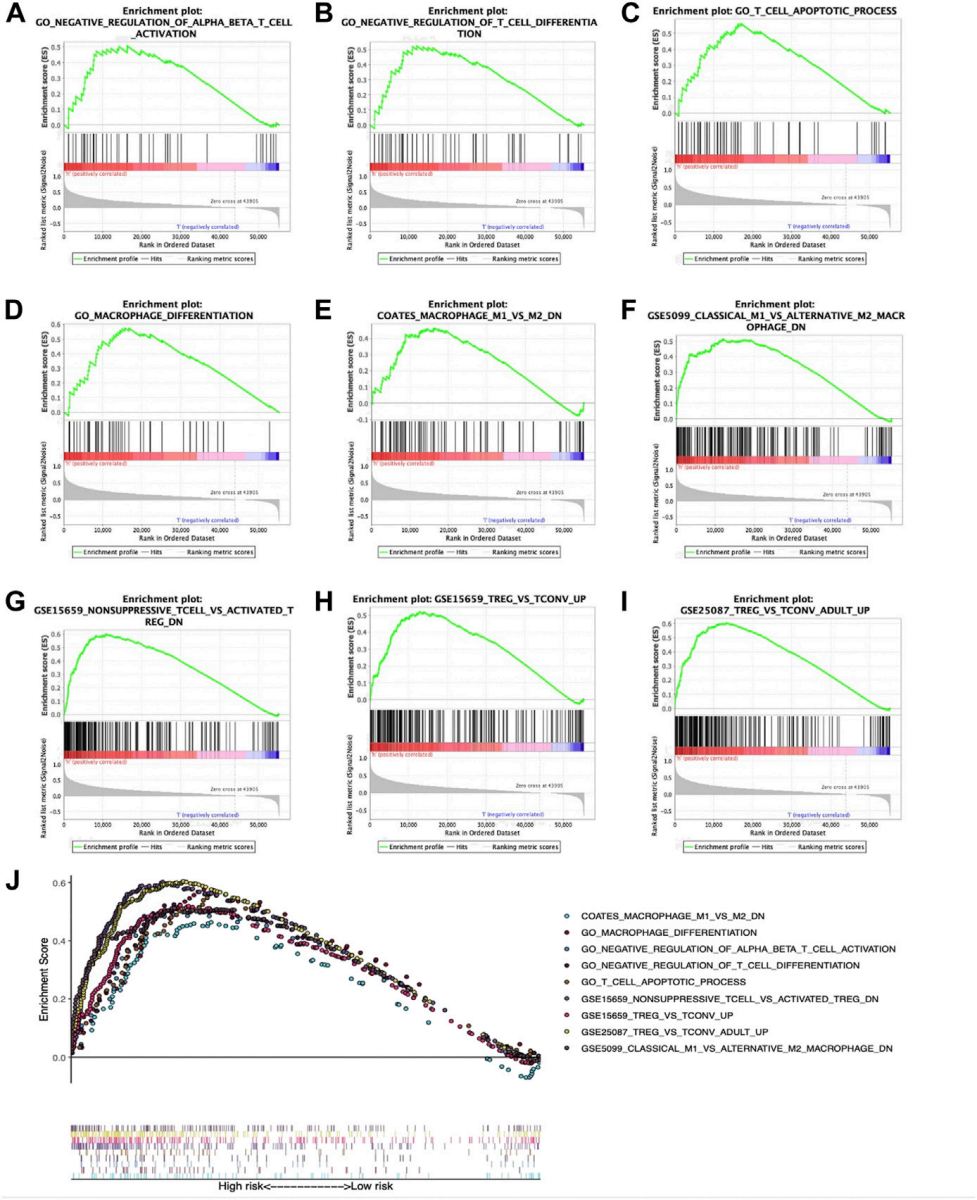
Enrichment plots showing GSEA-derived immune-related gene sets. Gene sets in **(A)** GO_αβ T-cell activation negative regulation, **(B)** GO_T-cell differentiation negative regulation, **(C)** GO_T-cell apoptotic process, **(D)** GO_macrophage differentiation, **(E)** COATES_macrophage M1 vs. M2 down, **(F)** GSE5099_classical M1 vs. alternative M2 macrophage down, **(G)** GSE15659_nonsuppressive T cell vs. activated Treg down, **(H)** GSE 15659_Treg vs. Tconv up, and **(I)** GSE25087_Treg vs. Tconv adult up were markedly associated with high-risk phenotype. **(J)** Summarization of those nine gene sets.

Relations of prognostic features with TIIC degrees among TCGA-HCC patients were analyzed to investigate whether risk score could be adopted for reflecting tumor microenvironment (TME) status. As a result, several immune-related gene sets, such as GO_T-cell differentiation negative regulation, GO_αβ T-cell activation negative regulation, GO_macrophage differentiation, GO_T-cell apoptotic process, COATES_macrophage M1 vs. M2 down, GSE5099_classical M1 vs. alternative M2 macrophage down, GSE15659_nonsuppressive T cell vs. activated Treg down, GSE25087_Treg vs. Tconv adult up, and GSE 15659_Treg vs. Tconv up, were also enriched, as revealed by GSEA (Figures 7A–I). A comprehensive diagram presenting the aforementioned items is shown in Figure 7J.

**FIGURE 8:**
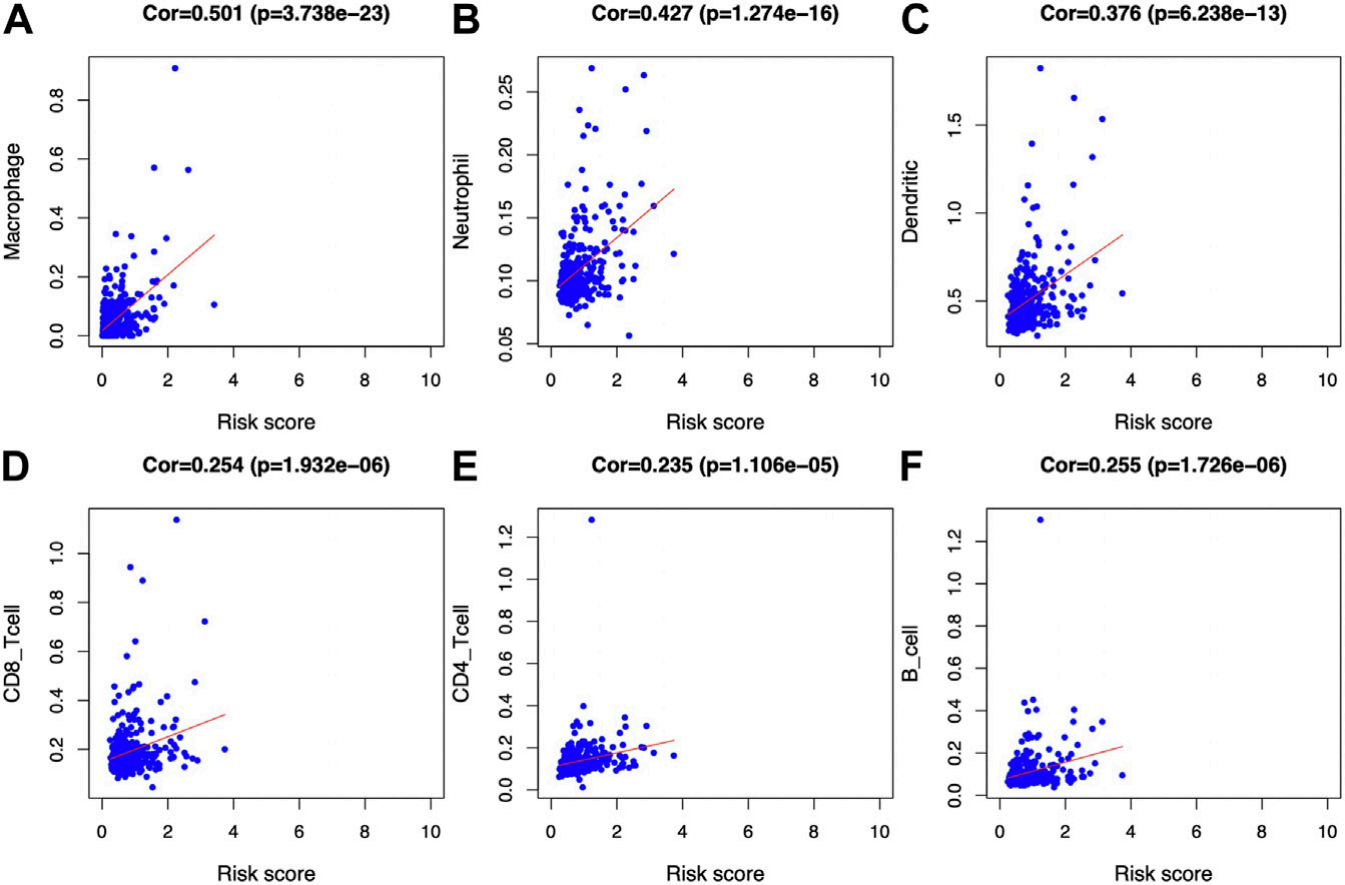
Associations of glycolysis with immune-associated prognosis prediction model and TIIC abundances. The associations were analyzed through PCA. **(A)** Macrophage; **(B)** neutrophil; **(C)** DC; **(D)** CD8+T cell; **(E)** CD4+T cell; and **(F)** B cell abundances.

**FIGURE 9:**
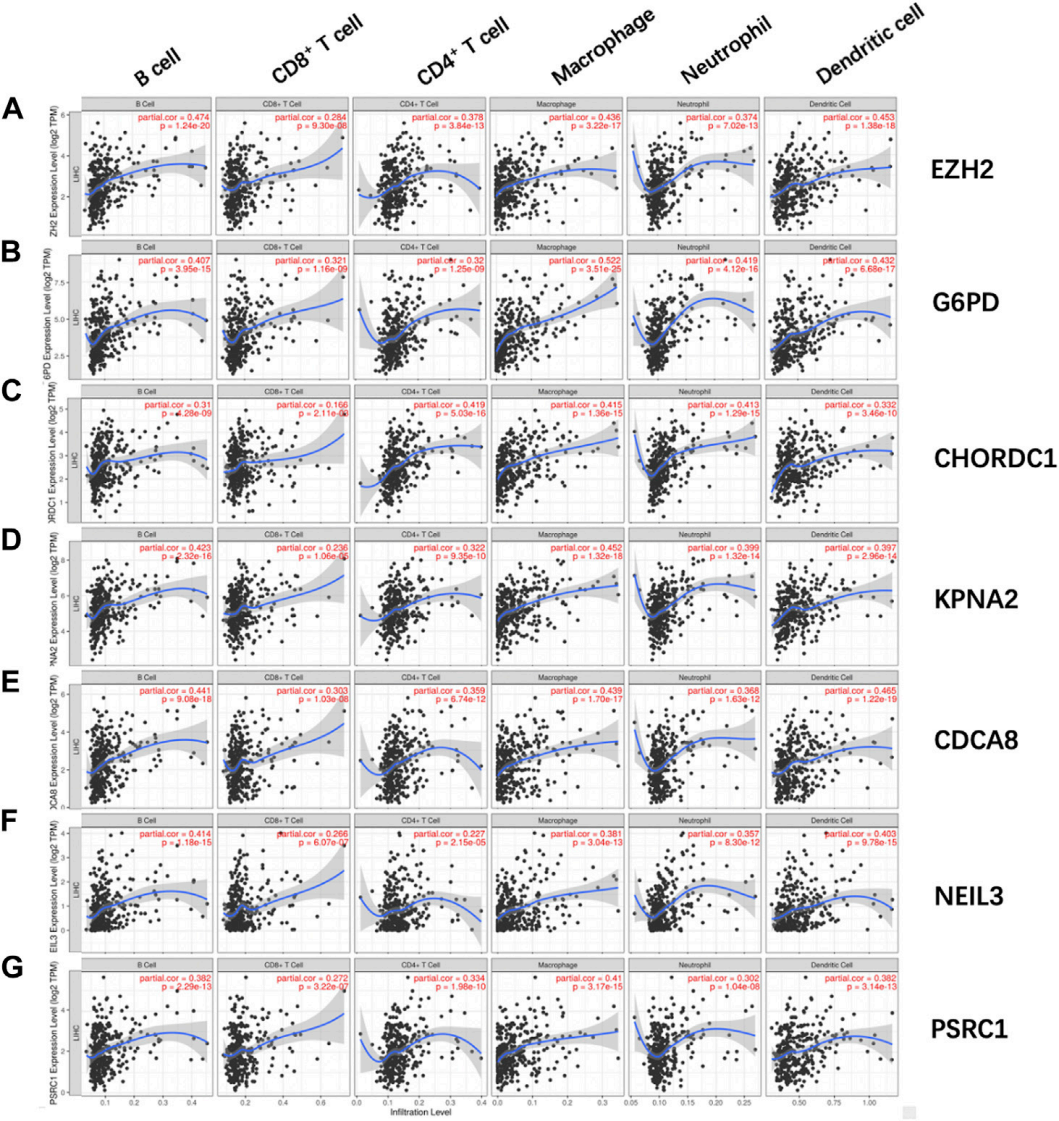
Relations of **(A)** *EZH2*, **(B)** *G6PD*, **(C)** *CHORDC1*, **(D)** *KPNA2*, **(E)** *CDCA8*, **(F)** *NEIL3*, and **(G)** *PSRC1* with the six kinds of TIICs, including macrophages, neutrophils, DCs, CD8 T cells, CD4 T cells, and B cells. *EZH2,* enhancer of zeste 2 polycomb repressive complex 2 subunit; *G6PD*, glucose-6-phosphate dehydrogenase; *CHORDC1*, cysteine and histidine-rich domain (CHORD) containing 1; *NEIL3*, nei endonuclease VIII-like 3; *PSRC1*, proline/serine-rich coiled-coil 1; *CDCA8*, cell division cycle-associated *8*; *KPNA2,* karyopherin alpha 2.

Afterward, the abundances of macrophages (Cor = 0.501; *p* = 3.738e−23), DCs (Cor = 0.376; *p* = 6.238e−13), and neutrophils (Cor = 0.427; *p* = 1.274e−16) markedly elevated within the TME of high-risk group (Figures 8A–C), indicating the diverse immune statuses between both groups. Additionally, CD4^+^ T cells (Cor = 0.235; *p* = 1.106e−05), CD8^+^ T cells (Cor = 0.254; *p* = 1.932e−06) (Figures 8D, E), and B cells (Cor = 0.255; *p* = 1.726e−06) (Figure 8F) showed low correlation in high-risk patients. In addition, the correlations between the seven model genes and the earlier mentioned six kinds of TIICs were analyzed based on the TIMER database (Figure 9).

**FIGURE 10:**
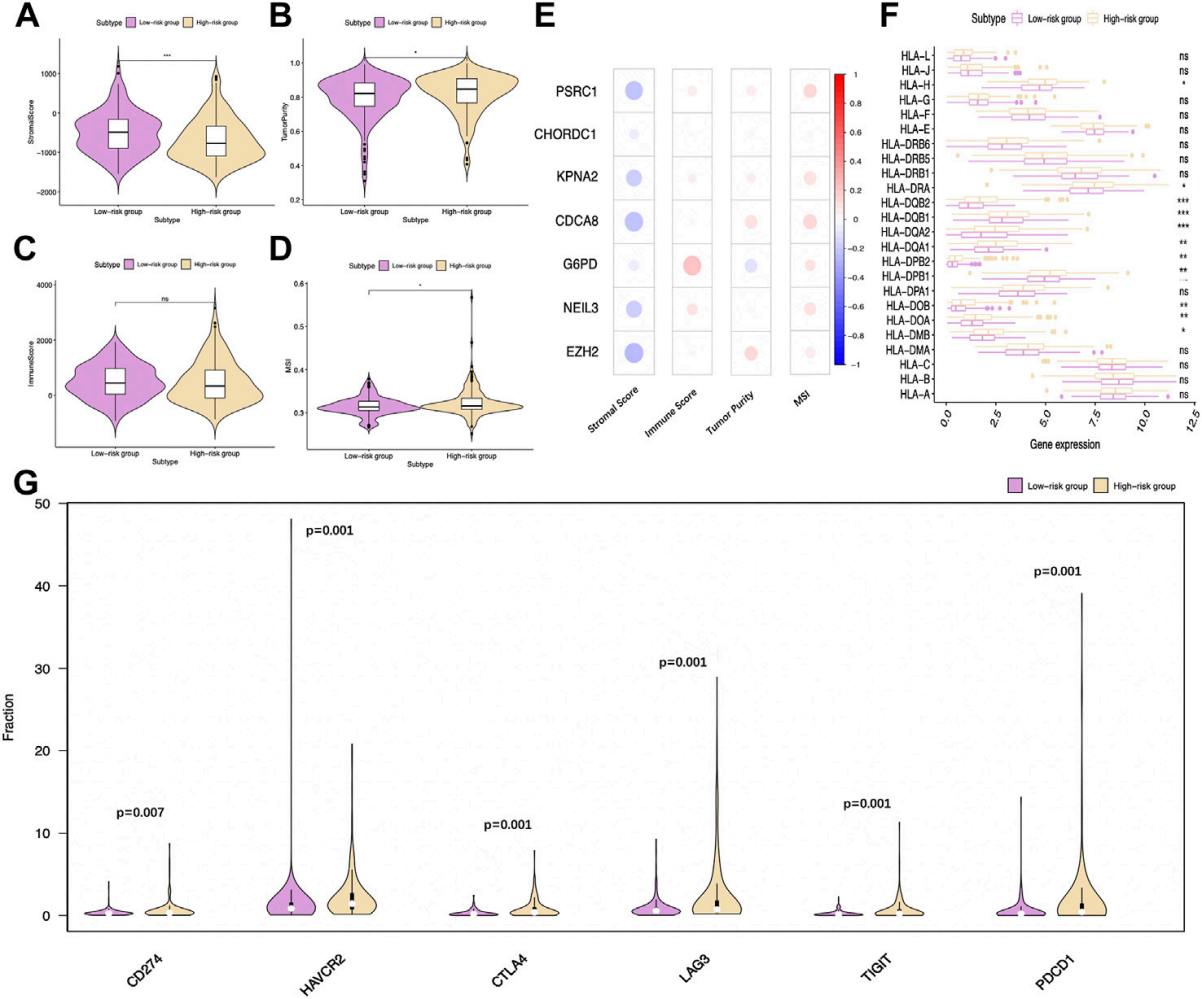
Distinct immune statuses between two TCGA-HCC cohorts. Comparisons on **(A)** stromal score, **(B)** tumor purity, **(C)** immune score, and **(D)** MSI of two groups. **(E)** Relations of the seven model genes with stromal score, immune score, tumor purity, and MSI are also exhibited. **(F)** Comparisons on HLA gene expression between two groups. **(G)** Differentially expressed immune checkpoints between two groups. CTLA-4: cytotoxic T-lymphocyte-associated protein 4; PD-1 (PDCD1): programmed cell death-1; TIM-3 (HAVCR2): T-cell immunoglobulin mucin receptor 3; PD-L1 (CD274): programmed death ligand 1; LAG3: lymphocyte activation gene-3; TIGIT: T cell immunoreceptor with Ig and ITIM domains.

Subsequently, ESTIMATE was conducted; as a result, the stromal scores of high-risk patients significantly decreased relative to low-risk patients, while tumor purity scores increased relative to the latter (Figures 10A,B). However, the difference in immune scores was not significant between the two groups (Figure 10C). Notably, the microsatellite instability (MSI) was elevated in the high-risk group compared to the low-risk group (Figure 10D). The relations of the seven model genes with immune score, stromal score, tumor purity, and MSI are shown in Figure 10E. Notably, some genes of human leukocyte antigen (HLA) were markedly upregulated among high-risk patients compared with low-risk patients (*p* < 0.001), including HLA-DQB2, HLA-DQA1, and HLA-DQA2 (Figure 10F).

Immunotherapy is becoming a well-recognized tumor treatment, which has improved the prognosis of diverse cancer cases. Therefore, this study analyzed the expression of common immune checkpoints in TCGA-derived HCC cases. The results suggested that, compared with low-risk patients, PD-1, PD-L1, lymphocyte activation gene-3 (LAG3), CTLA-4, T cell immunoreceptor with Ig and ITIM domains (TIGIT), and T-cell immunoglobulin and mucin-domain containing-3 (TIM-3) levels markedly increased in high-risk patients (Figure 10G). According to the earlier mentioned outcomes, the immunosuppressive environment among high-risk patients might predict a dismal prognostic outcome.

### Immunohistochemistry results regarding the expression of model genes in hepatocellular carcinoma tissues

**FIGURE 11:**
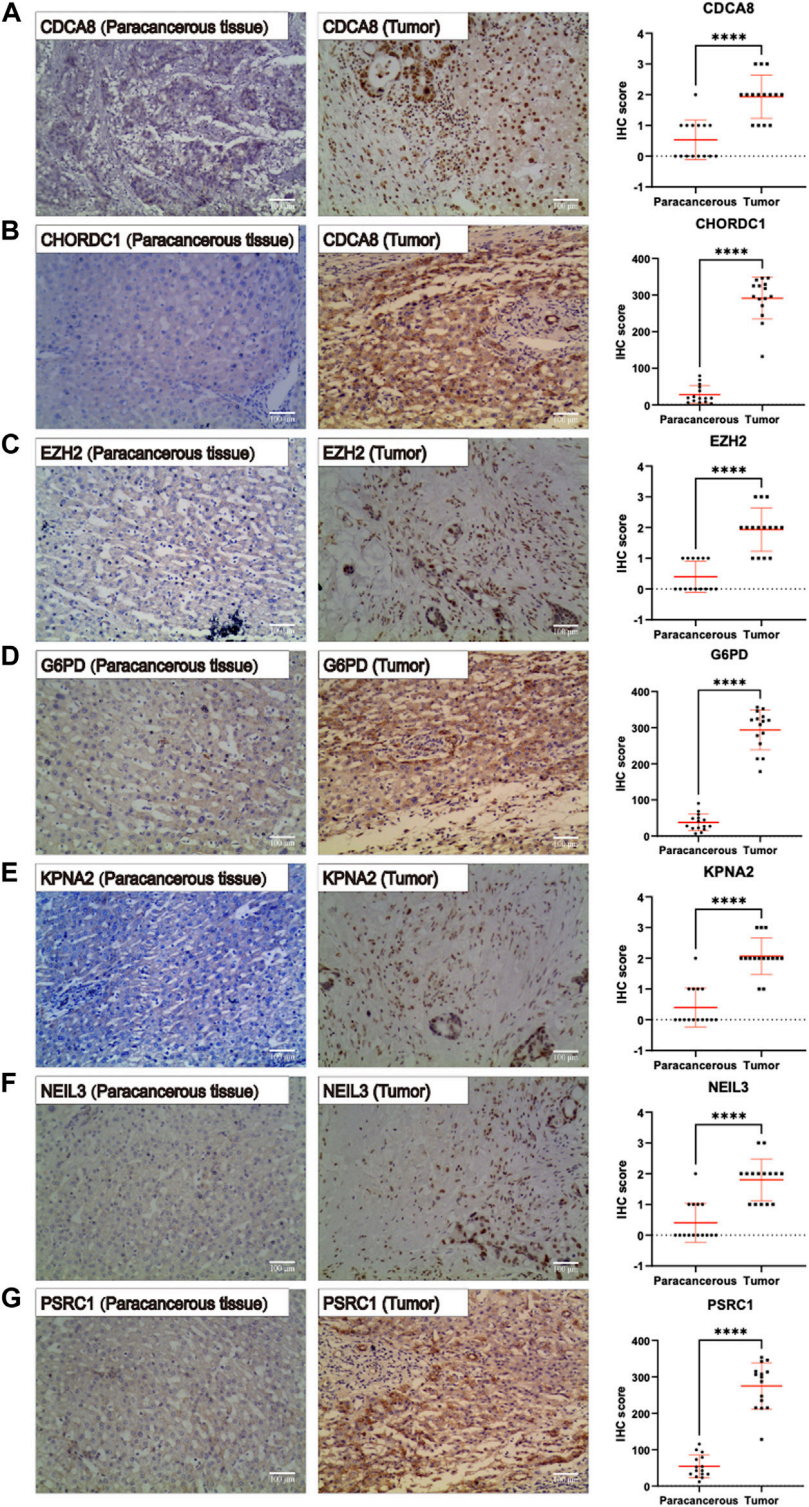
Immunohistochemistry pictures (200x, scale bar indicated 100 μm) show the protein expression of **(A–G)** *PSRC1*, *CHORDC1*, *KPNA2*, *CDCA8*, *G6PD*, *NEIL3*, and *EZH2* were remarkably enhanced in hepatocellular carcinoma (HCC) tissues relative to that in normal hepatic tissues.

IHC was carried out to examine the protein expression of seven signature genes, *PSRC1, CHORDC1, KPNA2, CDCA8, G6PD, EZH2,* and *NEIL3,* in 15 pairs of HCC tissues and their counterparts. It was discovered that the protein expression of all the signature genes was upregulated in HCC tissues relative to that in normal hepatic tissues (*p* < 0.001) (Figures 11A–G, respectively). This is consistent with the trend in risk coefficients for the model genes in our analysis.

## Discussion

Previous studies have demonstrated that the elevated glycolysis level, which is the tumor biomarker in cells, facilitates cancer cell survival under different conditions (Hamaguchi et al., 2008). Peng *et al.* reported that the abnormally expressed glycolytic enzymes, in particular the type-II hexokinase (HKII) and aldolase B (ALDOB), were associated with advanced HCC, dismal prognostic outcome, and early tumor recurrence (ETR). In addition, the downregulated expression of ALDOB in stage II HCC predicted ETR and the dismal outcome (Peng et al., 2008). Recent research also points out that, for HCC patients, the peritumoral monocytes will likely enhance the aerobic glycolytic level, while the aerobic glycolysis can thereby elicit the PD-L1 level *via* the PFKFB3-NF-κB pathway (Chen et al., 2019). Targeting aerobic glycolysis by dichloroacetate (DCA, an inhibitor of the pyruvate dehydrogenase kinase (PDK)) has been reported to improve the Newcastle disease virus (NDV)-regulated viro-immunotherapy for HCC, which is achieved through enhancing viral replication and alleviating the negative feedback of immunity (Meng et al., 2020). Therefore, research on immunity and glycolysis of HCC has been a research hot spot at present.

In the present work, complex bioinformatics analysis was conducted, and as a result, seven glycolysis-associated genes associated with HCC prognosis, including *PSRC1, CHORDC1, KPNA2, CDCA8, G6PD, NEIL3,* and *EZH2*, were identified*.* First, t-SNE, the machine-learning algorithm, offers a fine-grained and efficient way to reduce dimension, thus helping to explore potential prostate cancer (PC) (Ahmed et al., 2018) and breast cancer (BC) subtypes (Guo et al., 2019). In this study, t-SNE was adopted for identifying differential glycolytic patterns based on 200 glycolytic marker genes. Afterward, two clusters with different glycolysis statuses and prognostic outcomes were identified, and DEGs between the two groups were also analyzed. In addition, a glycolysis-associated risk score profile was constructed using those seven genes identified. Notably, our constructed model accurately distinguished high-from low-risk groups. In addition, low-risk HCC cases were proved to have prolonged OS compared with high-risk cases from the ICGC-HCC cohort. With regard to clinical utility, our constructed prognosis model showed significant relation to tumor grade among TCGA-HCC cases, as well as clinical stage, intrahepatic vein invasion, and portal vein invasion among ICGC-HCC cases. This indicated that the as-constructed model predicted a significantly higher risk among patients of advanced stage and grade. In addition, enhanced glycolytic activity is tightly related to aggressive clinicopathological characteristics of HCC, such as vascular invasion or portal vein tumor thrombosis (PVTT) (Liu et al., 2016). Moreover, PCA further validated the robust stratification ability of our model. In addition, the prognostic characteristics constructed in this study might predict the different prognostic outcomes of two TCGA groups stratified by tumor grade (G1+G2 or G3+G4), sex (male or female), AFP content (< 20 or ≥ 20 ng/ml), age (> 60), T stage (T1+T2) and clinical stage (I + II). Thus, the identified glycolysis-associated features might be related to HCC occurrence and development, making it a potentially valuable clinical biomarker. In addition, IHC validated the differential protein expression of model genes between HCC tissues and normal liver tissues adjacent to the carcinoma.

As a glycolysis-associated prognosis prediction signature, the high-risk patients showed a significantly enhanced glycolysis level compared with low-risk cases, as obtained from critical gene levels related to the glycolytic process. Aerobic glycolysis has been extensively reported as a potential predictive biomarker of HCC (Leung et al., 2015; Lu et al., 2017). Inhibiting aerobic glycolysis through blocking lactic dehydrogenase (LDH) can be adopted to treat HCC (Fiume et al., 2010). Also, our signature showed a positive relation to the abundance of six TIIC types, especially macrophages, neutrophils, and DCs. In addition, the results of GSEA suggested that T-cell differentiation and function in high-risk patients were impaired, while the proportion of Treg increased, and more macrophages seemed to transform into the M2 subtype, which might induce the immunosuppressive microenvironment. It is now well accepted that Tregs exist and are important in the control of immunological disorders (Baecher-Allan and Hafler, 2004; Sakaguchi, 2004). In particular, the TGF-β-miR-34a-CCL22 pathway-mediated Tregs are shown to enhance the venous transfer of HBV-positive HCC, associated with HCC metastasis and development (Yang et al., 2012). As revealed by Shen and colleagues, Tregs prevalence and extensive activities within the TME of HCC were related to the cancer stage, contributing to tumor flourishing and growth (Shen et al., 2010). Notably, in the setting of HCC, hypoxia-induced chemokine (C-C pattern) ligand 28 (CCL28) promotes Treg recruitment, leading to enhanced angiogenesis and VEGF expression (Facciabene et al., 2011; Ren et al., 2016). Moreover, Tregs exhibit promoted glycolysis, and glycolysis is required for the migration of Tregs, which is regulated by enzyme glucokinase *via* the PI3K-mTORC2 pathway (Kishore et al., 2017; Cluxton et al., 2019). As discovered by Rosa *et al.*, Tregs induction and suppression were mostly determined by glycolysis, while the latter regulated the Foxp3 splicing variants that contained exon 2 (Foxp3-E2) *via* the glycolytic enzyme enolase-1 (De Rosa et al., 2015). In terms of macrophages, the alternatively activated (M2) macrophages have been reported to exert an immunomodulatory effect, participate in the responses of polarized Th2 cells, and contribute to cancer progression (Shirabe et al., 2012). It is also elaborated that M2 macrophages are associated with the dismal prognostic outcome of HCC, which enhances cancer invasion *via* the C-C motif chemokine 22 (CCL22)-induced epithelial–mesenchymal transition (EMT) (Yeung et al., 2015). Glycolysis is also suggested to be engaged in the production of cytokines (like IL-6) by M2 macrophages (Chiba et al., 2017).

In addition, this work also explored the expression of immune checkpoints in two groups. According to our results, the levels of PD-1, PD-L1, LAG3, TIM-3, and CTLA-4 were remarkably elevated in the high-risk HCC group relative to the low-risk HCC group (*p* < 0.05). Based on the previously mentioned findings, the anti-immune checkpoint antibody treatment, which includes ipilimumab (the antibody against CTLA-4) and nivolumab (the antibody against anti-PD1) (Yang et al., 2019), can facilitate the treatment of high-risk patients compared with low-risk patients. Interestingly, our analysis confirmed the greater MSI among high-risk patients compared with low-risk ones. Previous studies have speculated that there may be a link between MSI itself and glycolytic activity (Chung et al., 2013). Furthermore, it is recently been suggested that MSI-high (MSI-H) cancers, despite the primary sites, can favorably respond to ICIs (Eso et al., 2020). According to the aforementioned results, high-risk cases identified using our model were more suitable for ICIs therapy.

However, certain limitations should be mentioned in the current study. First, the influence of pretreatments among HCC cases, such as transarterial chemoembolization and hepatic resection, on glycolysis status and immune components remained unknown, because of the lack of clinical data. Moreover, large prospective studies and additional *in vivo* and *in vitro* experimental studies are still needed to confirm our findings.

## Conclusion

In summary, this work establishes the prognosis prediction model and verifies it using six glycolysis-associated genes, which can be adopted for predicting the OS of HCC. The constructed prognosis model facilitates the selection of personalized treatment strategies in the clinic. In addition, the prognosis signature is associated with glycolysis and immune status, which helps to comprehensively clarify the mechanism related to HCC prognosis.

## Data Availability

The datasets presented in this study can be found in online repositories. The names of the repository/repositories and accession number(s) can be found in the article/Supplementary Material.
