# Supplementary figures and images for: Development and verification of the glycolysis-associated and immune-related prognosis signature for hepatocellular carcinoma
Source: Front Genet. 2022 Oct 4;13:955673. doi: 10.3389/fgene.2022.955673 (PMC9576873; doi:10.3389/fgene.2022.955673)

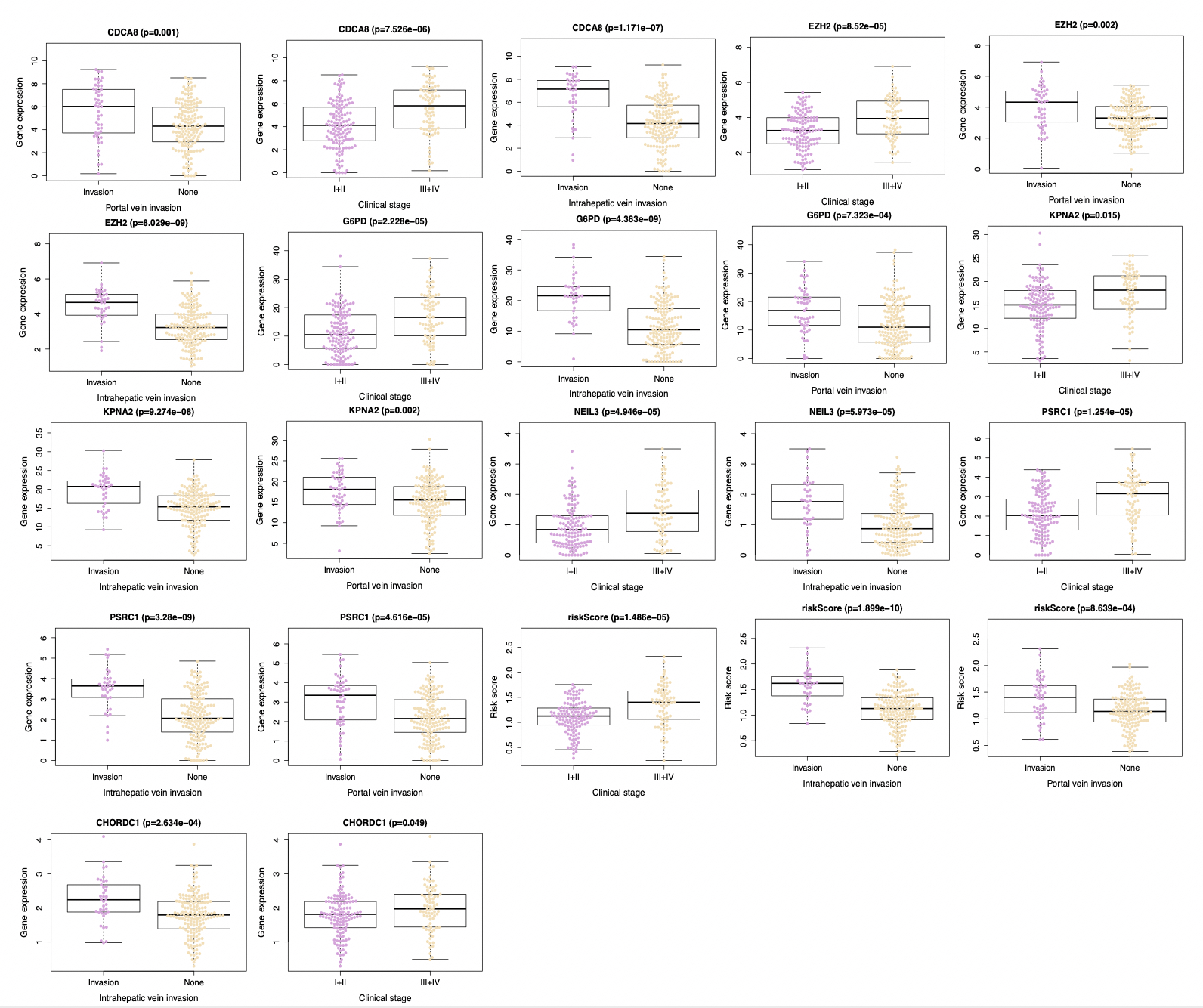

Supplement: Supplementary file 3 [file Image1.TIFF]
